# Burnout Syndrome amongst Medical Students in Cameroon: A Cross-Sectional Analysis of the Determinants in Preclinical and Clinical Students

**DOI:** 10.1155/2019/4157574

**Published:** 2019-01-03

**Authors:** Tsi Njim, Haman Makebe, Louise Toukam, Belmond Kika, Steve Fonkou, Johnson Fondungallah, Azingala Fondong

**Affiliations:** ^1^Health and Human Development (2HD) Research Network, Douala, Cameroon; ^2^Department of Paediatrics and Internal Medicine, Faculty of Health Sciences, University of Buea, Buea, Cameroon; ^3^Faculty of Health Sciences, University of Bamenda, Bamenda, Northwest Region, Cameroon; ^4^District Hospital Ekondo-Titi, Ekondo-Titi, Southwest Region, Cameroon; ^5^Buea Town Medical Centre, Buea, Southwest Region, Cameroon

## Abstract

**Background:**

Due to a series of recognised psychological stressors in the traditional path of medical studies, medical students are prone to experience burnout syndrome.

**Objective:**

This study aimed to determine the predictors of burnout syndrome amongst medical students in Cameroon.

**Methods:**

This was a cross-sectional study which recruited 413 medical students consecutively after sampling three of the five medical schools in Cameroon using a random sequence generator. Data were collected via a printed self-administered questionnaire from consenting participants assessing burnout syndrome using the OLdenburg Burnout Inventory (OLBI). Data was analysed using Stata version 12 and p value significance was set at 95%. Multivariable linear regression was used to identify independent determinants of burnout syndrome.

**Results:**

An alpha Cronbach coefficient of 0.74 showed that the OLBI assessed the same underlying construct of burnout syndrome in this population. The results of the multiple linear regression showed that five predictors explained 35.0% of the variance (R^2^= 35.0, F(9, 184) = 10.99, p <0.001). It was found that marital status significantly predicted burnout (Beta: 4.82, p value: 0.024), as well as relationship difficulties (Beta: 3.17, p value <0.001), cumulative GPA (Beta: -2.15, p value: 0.006), regretting the choice of medical studies (Beta: 7.85, p value <0.001), and recreational drug use (Beta: 6.99, p value: 0.005).

**Conclusion:**

Early detection of burnout in medical students in Cameroon could be done by identifying and addressing the potential determinants. The institution of preventive measures against burnout syndrome in medical schools in Cameroon is warranted to decrease the morbidity associated with the condition.

## 1. Background

Burnout syndrome is a psychological state resulting from prolonged exposure to job stressors which has been studies by several scholars in healthcare and other fields [1–4]. As shown by several models developed in many years to explain burnout syndrome and recently reviewed by Chirico [5], this syndrome results from an imbalance between high job demands and low resources [6–8]. It is agreed amongst most scholars that individuals suffering from burnout usually experience high levels of emotional exhaustion and depersonalization or disengagement [9, 10]. Medical students are constantly exposed to psychological stressors with distinct and recognised events in their academic life acting as potential determinants that could lead to a phenomenon of “psychological toxicity” [11, 12]. The highly competitive entrance exams to get into medical school in Cameroon coupled with difficult transitions from the preclinical to the clinical phase, the reality of facing critically ill patients with dire prognosis, problems associated with newfound autonomy in university life, and the excessive course and clinical workload are traditional stressful patterns encountered in the life of most medical students [12]. This is compounded by the sheer lack of mental health assistance for medical students and other health personnel.

The prevalence of burnout syndrome amongst medical personnel is generally high, ranging from 27% to 75% [13–15], and it is recognised that the syndrome begins at the early stages of medical training due to the aforementioned stressors and often worsens during practice with increased workload [16–19]. This is particularly worrisome in Cameroon which has a population of about 24 million and a physician density of approximately 1: 12,500 people [20, 21] with this density increasing to about 1:26,726 persons in some regions [22]. Furthermore, burnout has been associated with an increased risk of depression [23]. A recent study in the four medical schools in Cameroon showed that 30.6% of medical students had a major depressive disorder [24]. There might therefore be a high prevalence of burnout amongst these students which could have consequences for patient outcomes and health institutions in the country due to the potential increased workload as demonstrated by the physician density and adverse working conditions in this setting [25]. There is scarcity of research on burnout amongst medical students in Cameroon.

This research will therefore help to encourage early detection of burnout syndrome in medical students such that appropriate preventive measures may be instituted. It could also help inform public policy and add to the current knowledge of the state of mental health amongst medical students in Cameroon.

## 2. Methods

### 2.1. Population and Design

#### 2.1.1. Design

This manuscript is part of the Me-HeLTC project. This was a cross-sectional study that attempted to assess burnout and depression amongst medical students in Cameron employed in a period of three months (January 2018–March 2018); we followed similar methods used by Njim et al., 2018 [26].

#### 2.1.2. Population, Sampling, and Sample Size

The sampling frame included all the medical students in the five recognised medical schools in Cameroon which have students in the clinical phases of study: University of Yaounde I, University of Douala, University of Buea, University of Bamenda, and Université des Montagnes. The following sampling strategy was used: The five universities were assigned whole integers from one to five and a random sequence generated. The first three universities (University of Bamenda, University of Douala, and University of Buea) were selected. From each selected university, at least 80 consenting medical students were recruited by a consecutive convenience sampling until the sample sign as calculated by the formula below was attained [27]:

(1) $$\begin{array}{c} n=\frac{Z^{2}P\left(1-P\right)}{d^{2}} \end{array}$$

where n is the sample size, Z is the Z statistic for a level of confidence, P is the expected prevalence or proportion (in proportion of one: 32%, P = 0.32) [21], and d is the precision (in proportion of one; if 5%, d = 0.05). Z statistic (Z): for the level of confidence of 95%, which is conventional, Z value is 1.96, for a 95% confidence intervals (CI). A minimum of 335 medical students were required for the study.

#### 2.1.3. Ethical Approval and Consent to Participate

Ethical approval was sought from the Cameroon Baptist Health Board Institutional Review Board and Administrative Authorization was obtained from the regional delegation of Public Health of the Southwest Region. All ethical principles were respected during the conduct of this research and confidentiality was assured by collection of anonymous data. All medical students who accepted to partake signed a written informed consent document.

Medical students from the selected universities were targeted and their written consent obtained. They were handed a printed questionnaire in either English or French depending on their language of preference which they filled at their convenience and handed in; with a total of 413 questionnaires returned from a possible 500, the response rate given was 82.6%.

#### 2.1.4. Instrument

Data was collected using a printed structured questionnaire where the following characteristics were collected:

Sociodemographic characteristics: age; sex; marital status; presence and number of (children) dependents; level of medical studies (preclinical and clinical); number of hours spent studying; presence of problems in personal relationships (defined as a close connection between two people formed by emotional and sexual interactions); monthly income (in United States Dollars) and the perception of sustainability on their monthly income; average academic results attained (measured by the cumulative grade point average); alcohol use (measured in units of alcohol); smoking; and history of any chronic disease (which included asthma, chronic pelvic pain, hypertension, diabetes mellitus, gastroesophageal reflux disease, chronic peptic ulcer disease, migraines, cancers, HIV/AIDS, cerebral lesions, chronic liver, and kidney disease). An open-ended question was also provided for the students to state their chronic illness which was used by the investigators to assess the suitability of the response for this scenario. These variables were selected after a literature review of potential predictors of burnout syndrome [1, 5, 12, 28, 29].

The next section assessed burnout syndrome using the OLdenburg Burnout Inventory (OLBI). The OLBI is a self-administered questionnaire consisting of 16 positively and negatively framed questions to assess two core parameters of burnout with eight questions for each parameter: exhaustion (intense physical, affective, and cognitive strain) and disengagement (characterized as cynicism referring to distancing from one's work in general) [10]. The questionnaire was adapted for use in an academic milieu [30]. The respondents answered the above questions according to perceived frequency of occurrence on a scale of one to four ranging from strongly agree to strongly disagree. Some of the exhaustion items included were “there are days when I feel tired before I arrive at work”; “after work, I tend to need more time than in the past in order to relax and feel better”; “I can tolerate the pressure of my work very well”; “during my work, I often feel emotionally drained”; and “after working, I have enough energy for my leisure activities”. Disengagement items included questions like “I always find new and interesting aspects in my work”; “it happens more and more often that I talk about my work in a negative way”; “lately, I tend to think less at work and do my job almost mechanically”; and “I find my work to be a positive challenge”.

The third section assessed depression using the reliable and valid 9-item self-administered Patient Health Questionnaire (PHQ-9) [31]. For the questions in the PHQ-9, participants were asked if over the last 2 weeks, they had been bothered by problems including “little interest or pleasure in doing things”; “feeling down, depressed, or hopeless”; “trouble falling or staying asleep, or sleeping too much”; “feeling tired or having little energy”; “poor appetite or overeating”; “feeling bad about yourself—or that you are a failure or have let yourself or your family down”; “trouble concentrating on things, such as reading the newspaper or watching television”; “moving or speaking so slowly that other people could have noticed? Or the opposite—being so fidgety or restless that you have been moving around a lot more than usual”; and “thoughts that you would be better off dead or of hurting yourself in some way”. Participants were expected to respond if these problems affected them “not at all”, “several days”, “more than half of the days”, or “nearly every day”.

### 2.2. Data Management and Statistical Analysis

Data entry was done using EPI Info version 7 (CDC, Atlanta). To ensure accurate data entry, a random sample of 10% of the data was crosschecked. Data back-up was done daily and data analysis was carried out using Stata software package version 12 (StataCorp, College Station, TX, USA).

Results were presented as count (percentages), mean, and standard deviation (SD) or median and 25^th^-75^th^ percentiles as appropriate. The reliability and validity of the OLBI were assessed by means of Cronbach alpha coefficients and factor analyses. Multivariable linear regressions were used to identify predictors of burnout amongst the participants. Significance was set up at p < 0.05.

The two subscales of the OLBI (emotional exhaustion and cynicism) were used to define burnout. Both subscales were added to obtain a total score for burnout syndrome. Negatively phrased questions were reversed (1=4, 2=3, 3=2, 4=1) before the average scores for each subscale were calculated such that higher scores indicate higher exhaustion and disengagement.

Univariable linear regression analyses were used to compare the outcome—burnout as a continuous variable with potential predictors such as marital status, sex, age, level of studies, presence of a chronic illness, poor academic performance, alcohol consumption, and smoking. Predictors significant at 95% were inputted in a multivariable linear regression model to identify independent predictors of burnout syndrome.

## 3. Results

### 3.1. General Characteristics

A total of 413 questionnaires were returned from the medical students after 500 were handed out giving a response rate of 82.6%. There were 172 (41.65%) students from the university of Buea and 183 (44.96%) students in total, from the clinical level. A majority (54.61%) of the students were female and most of them (97.04%) were single (Table 1). The ages of the students ranged from 16 to 30 years with a mean age of 21.21 ± 2.43 years and the average number of hours spent studying was 3.82 ± 0.11 hours (Table 2).

### 3.2. Assessment of Burnout Syndrome

The average for disengagement items was 16.64 ± 3.39 (range 9–31) while the average for exhaustion items was 20.49 ± 3.53 (range 11–32). All the items showed high covariances and the alpha Cronbach coefficient was 0.74 showing that the OLBI measured the same underlying construct of burnout amongst all participants (Additional File 1).

### 3.3. Determinants of Burnout Syndrome

On univariable linear regression analysis, burnout syndrome was associated with marital status [regression coefficient (RC): 6.19, 95% confidence intervals (CI): 2.82, 9.56, p value: <0.001]; relationship difficulties (RC: 2.55, 95% CI: 1.05, 4.05, p value: 0.001); number of children (RC: 2.47, 95% CI: 0.52, 4.41, p value: 0.013); level of studies (RC: 2.05, 95% CI: 0.912, 3.20 p value: <0.001); number of hours studying (RC: -0.42, 95%CI: -0.69, -0.15, p value: 0.003); sufficient monthly income (RC: -1.45, 95% CI: -2.65, -0.26, p value: 0.017); cumulative grade point average (GPA) (RC: -1.48, 95% CI: -2.85, -0.11, p value: 0.034); regret choice of medical studies (RC: 7.19; 95% CI: 5.23, 9.16, p value: <0.001); and recreational drug use (RC: 4.39, 95% CI: 0.50, 8.28, p value: 0.027) (Table 3).

A multiple regression was calculated to predict levels of burnout based on the sociodemographic data. The results of the multiple linear regression showed that five predictors explained 35.0% of the variance (R^2^ = 35.0, F(9, 184) = 10.99, p <0.001). It was found that marital status significantly predicted burnout (Beta: 4.82, p value: 0.024), as well as relationship difficulties (Beta: 3.17, p value <0.001), cumulative GPA (Beta: -2.15, p value: 0.006), regretting the choice of medical studies (Beta: 7.85, p value <0.001), and recreational drug use (Beta: 6.99, p value: 0.005) (Table 4).

## 4. Discussion

In this study, we assessed the determinants of burnout syndrome in medical students in Cameroon and found out that independent predictors of burnout in this population were to be married, difficulties in personal relationships, a low cumulative GPA, regret in choosing medical studies, and recreational drug use.

The sociodemographic characteristics usually associated with burnout include sex and age [29] though the direction of this correlation remains unclear [5]. In this study, however, we found out that those who were married were more likely to have higher burnout scores. Medical studies are highly loaded with psychological stressors and the addition of family-work conflict could lead to students having a higher risk of becoming burned out. This was confirmed by the fact that those who admitted having strains and difficulties in their personal relationships had about three units more of burnout on the OLBI scale.

Students who regretted choosing medical studies had higher burnout scores than those who were comfortable with medicine as a chosen career path. Several studies have shown an association between considering abandoning the medical course or felt uncomfortable with the course activities and burnout [12, 28, 29]. Individuals who are uncomfortable with their choice of studies can gradually start resenting the course as they feel inefficacious in their academic environment [29]. Academic activities then become more stressful leading to a toxic academic milieu [29] making them more prone to burnout. This situation could become compounded if this stress is reflected in the results of the students. As the student becomes more stressed, poor results may then follow leading to more self-doubt and a vicious cycle of stress, poor results, self-doubt, and decrease efficacy. Indeed, in this study, we found out that increase in the cumulative GPA (a university grading system on a total of four) was negatively correlated with burnout syndrome. This showed that higher performing students were less likely to be burned out than underperforming students.

In a similar study we carried out in 2018 amongst nursing students, it was also shown that regret of choice of nursing studies was an independent predictor of burnout in this population [26]. Also, it was shown in this same study that dissatisfaction with results amongst the nursing students was associated with burnout [26]. This dissatisfaction with results could arise as a result of poor performance in studies. This reaffirms the hypothesis that the stressful nature of academic studies in this domain could lead to a vicious cycle of decreased efficacy, poor performance, and burnout.

Recreational drug use was also found to be independently associated with burnout syndrome, with students who used recreational drugs like marijuana and tramadol having higher burnout scores on the OLBI burnout scale. This is a unique and interesting finding, but it is uncertain whether recreational drug use directly results in students becoming more burned out or if students who are already underperforming and are experiencing high levels of stress turn to recreational drug use as a source of comfort [32]. This association has to be explored more in prospective study designs.

Burnout syndrome is increasingly being recognised as a significant mental health issue in global health around the world [13]. However, like several mental health disorders, this syndrome amongst others is highly neglected in developing countries and in Cameroon in particular [33, 34]. Medical students in Cameroon face significant psychological stressors which include a high course workload, issues with financial sustainability, and realities around an autonomous university life. This stressors could lead to burnout. Indeed, a recent study showed that a third of all medical students in Cameroon have a depressive disorder. The situation may get worse as these students leave medical school due to the low physician-patient ratio that awaits them, low remuneration, inability to practice with professional autonomy, limited career advancement opportunities, and limited appropriate equipment for effective practice: conditions which are prevalent in Cameroon [35].

The early identification of these potential determinants of burnout in medical students in Cameroon should therefore warrant referral to adequate psychiatric departments to reduce the morbidity associated with this mental health problem.

## 5. Limitations

Due to the cross-sectional design, temporal associations and causality cannot be ascertained. Also, multiple linear regression analysis can only ascertain associations and not causal relationships. The use of questionnaires also required that these participants had to recall events and characteristics in the past, making the study subject to recall bias.

However, this is the first study to assess the use of the OLBI inventory in this population and the predictors of burnout amongst Cameroonian medical students. The alpha Cronbach coefficient confirmed that this tool measures the same underlying construct in this population and is therefore reliable and valid for future use. This study will also add to the cartography of mental health issues in Cameroon. This will help inform policy makers on the need for early detection and initiation of preventive measures against burnout syndrome amongst students.

## 6. Conclusion

Burnout syndrome in medical students in Cameroon is associated with a married marital status, relationship difficulties, low cumulative GPAs, regret for choosing medical studies and recreational drug use. Early identification of this determinants is necessary to reduce the morbidity associated with this mental health problem and to carry out preventive measures by occupational stakeholders and policymakers.

## Figures and Tables

**Table 1:** Categorical variables showing the sociodemographic characteristics of 413 medical students in Cameroon assessed for burnout syndrome from January to March 2018.

**Variable**		**Preclinical level**		**Clinical level **		**Total **	
		**(n = 224)**		**(n = 183)**			
		n	%	n	%	N	%
Medical school (n = 413)	University of Bamenda	59	37.11	96	60.38	159	38.50
	University of Buea	86	50.00	85	49.42	172	41.65
	University of Douala	79	96.34	2	2.44	82	19.85

Gender (n = 412)	Male	81	43.32	104	55.61	187	45.39
	Female	143	63.56	79	35.11	225	54.61

Presently in a relationship (n = 411)	Yes	73	44.24	91	55.15	165	40.15
	No	150	60.98	92	37.40	246	59.85

Marital status (n = 406)	Single	216	54.82	173	43.91	394	97.04
	Married	3	25.00	9	75.00	12	2.96

Difficulties in personal relationship^a^ (n = 367)	Yes	30	38.96	47	61.04	77	20.98
	No	168	57.93	117	40.34	290	79.02

Exams re-sited (n = 404)	Yes	102	43.40	131	55.74	235	58.17
	No	116	68.64	50	29.59	169	41.83

Courses repeated (n = 399)	Yes	78	48.15	81	50.00	162	40.60
	No	133	56.12	102	43.04	237	59.40

Satisfaction with results (n = 379)	Yes	47	46.08	53	51.96	102	26.91
	No	147	53.07	127	45.85	277	73.09

Regret choice of medical studies (n = 409)	Yes	6	17.65	27	79.41	34	8.31
	No	215	57.33	156	41.60	375	91.69

Occurrence of life changing crises in last six months^b^ (n = 400)	Yes	68	43.04	88	55.70	158	60.50
	No	148	61.16	91	37.60	242	60.50

Presence of chronic illness^c^ (n = 411)	Yes	10	52.63	8	42.11	19	4.62
	No	214	54.59	174	44.39	392	95.38

Alcohol consumption (n = 410)	Yes	49	39.84	73	59.35	123	30.00
	No	173	60.28	110	38.33	287	70.00

Recreational drug use^d^ (n = 408)	Yes	8	88.89	1	11.11	9	2.21
	No	213	53.38	181	45.36	399	97.79

Sufficient monthly income (n = 393)	Yes	101	63.92	56	35.44	158	40.20
	No	107	45.53	125	53.19	235	59.80

^a^Personal relationship was defined as close connections between two people formed by emotional and sexual interactions. ^b^Life changing crises defined as loss of a loved one, physical or sexual trauma, and condition of emotional or social instability. ^c^Chronic illnesses included asthma, chronic pelvic pain, diabetes mellitus, gastroesophageal reflux disease, chronic peptic ulcer disease, migraines, cerebral lesions, and paralysis; ^d^recreational drugs included marijuana and tramadol.

**Table 2:** Continuous variables showing the sociodemographic characteristics of 413 medical students in Cameroon assessed for burnout syndrome from January to March 2018.

**Variable**	**Number of observations**	**Preclinical level (n = 224)**		**Clinical level (n = 183)**		**Total sample**			
		Mean	SD	Mean	SD	Mean	SD	Min	Max
Age	374	19.64	0.11	23.10	0.15	21.21	2.43	16	30

Number of hours studying	387	3.69	0.16	3.96	0.16	3.82	0.11	0	18

Monthly income	267	45.27	3.26	51.10	3.52	48.45	2.41	0	281.52

Quantity of alcohol consumed	137	0.57	0.14	0.84	0.20	0.72	1.53	0	15

GPA	311	2.79	0.05	2.90	0.03	2.85	0.49	0	4

USD: United States dollars; GPA: cumulative grade point average; OLBI: OLdenburg Burnout Inventory.

**Table 3:** Univariable linear regression analysis of potential determinants of burnout syndrome amongst 413 medical students who were assessed for burnout syndrome from January to March 2018 in Cameroon.

**Variables**	**Coefficient**	**Intercept**	**SE**	**95**%** CI**	**p value**
Age	0.20	32.86	0.13	-0.05, 0.45	0.119

Gender (Female/Male)	1.08	36.54	0.58	-0.06, 2.23	0.064

Marital status (married/single)	6.19	36.98	1.71	2.82, 9.56	<0.001

Difficulties in personal relationships (Yes/No)	2.55	36.66	0.76	1.05, 4.05	0.001

Number of children	2.47	36.86	0.99	0.52, 4.41	0.013

Level of studies (clinical/Preclinical)	2.05	36.17	0.58	0.912, 3.20	<0.001

Number of hours studying	-0.42	38.78	0.14	-0.69, -0.15	0.003

Monthly income	0.01	36.85	0.01	-0.01, 0.03	0.412

Sufficient monthly income (Yes/No)	-1.45	37.77	0.61	-2.65, -0.26	0.017

Cumulative GPA	-1.48	41.14	0.70	-2.85, -0.11	0.034

Regret choice of medical studies (Yes/No)	7.19	36.51	1.00	5.23, 9.16	<0.001

Recreational drug use (Yes/No)	4.39	37.06	1.98	0.50, 8.28	0.027

Presence of chronic illness (Yes/No)	0.01	37.15	0.39	-2.72, 2.74	0.994

**Table 4:** Multivariable linear regression analysis of potential determinants of burnout syndrome amongst 413 medical students who were assessed for burnout syndrome from January to March 2018 in Cameroon.

**Variables**	**Coefficient**	**SE**	**95**%** CI**	**p value**
Marital status (married/single)	4.82	2.12	0.63, 9.01	0.024
Relationship difficulties (Yes/No)	3.17	0.88	1.44, 4.90	<0.001
Number of children	-0.58	1.02	-2.58, 1.43	0.570
Level of studies (Clinical/Preclinical)	1.27	0.80	-0.32, 2.85	0.117
Number of hours studying	0.02	0.22	-0.40, 0.45	0.922
Sufficient monthly income (Yes/No)	-0.20	0.81	-1.79, 1.39	0.803
Cumulative GPA	-2.15	0.78	-3.68, -0.62	0.006
Regret choice of medical studies (Yes/No)	7.85	1.38	5.12, 10.57	<0.001
Recreational drug use (Yes/No)	6.99	2.48	2.10, 11.88	0.005
Intercept	40.50	2.33	35.91, 45.08	<0.001

## Data Availability

The datasets used and/or analysed during the current study are available from the corresponding author on reasonable request.

## Abbreviations

OLBI: OLdenburg Burnout Inventory
GPA: Grade point average
SE: Standard error
SD: Standard deviation
CI: Confidence interval.

## Supplementary Materials

Supplementary Materials Additional File 1: table showing the calculation of interitem covariances of the 16-item OLBI inventory assessing burnout in two subscales (disengagement and exhaustion) and the alpha Cronbach coefficients. All items show high covariances, and the alpha Cronbach coefficient of 0.74 shows that the inventory assesses the same underlying construct of burnout syndrome amongst this population. Items assessing disengagement are marked with (D) while items assessing exhaustion are marked with (E). Items which were reversed before analysis are marked with (R).
