# Supplementary material for: Burnout Syndrome amongst Medical Students in Cameroon: A Cross-Sectional Analysis of the Determinants in Preclinical and Clinical Students
Source: Psychiatry J. 2019 Jan 3;2019:4157574. doi: 10.1155/2019/4157574 (PMC6335680; doi:10.1155/2019/4157574)
Supplement: Supplementary Materials — Additional File 1: table showing the calculation of interitem covariances of the 16-item OLBI inventory assessing burnout in two subscales (disengagement and exhaustion) and the alpha Cronbach coefficients. All items show high covariances, and the alpha Cronbach coefficient of 0.74 shows that the inventory assesses the same underlying construct of burnout syndrome amongst this population. Items assessing disengagement are marked with (D) while items assessing exhaustion are marked with (E). Items which were reversed before analysis are marked with (R). [file 4157574.f1.docx]

| **Item** | **Item-test correlation** | **Item-rest correlation** | **Average inter-item covariance** | **Alpha Cronbach coefficient** |
| --- | --- | --- | --- | --- |
| I always find new and interesting aspects in my studies (D) | 0.47 | 0.39 | 0.11 | 0.73 |
| It happens more and more often that I talk about my studies in a negative way (D) (R) | 0.58 | 0.48 | 0.10 | 0.72 |
| Lately, I tend to think less about my academic tasks and do them almost mechanically (D) | 0.44 | 0.31 | 0.11 | 0.73 |
| I find my studies to be a positive challenge (D) | 0.37 | 0.26 | 0.11 | 0.74 |
| Over time, one can become disconnected from this type of studies (D) (R) | 0.48 | 0.35 | 0.10 | 0.73 |
| Sometimes I feel sickened by my studies (D) (R) | 0.58 | 0.46 | 0.10 | 0.72 |
| Medicine is the only field of study that I can imagine myself doing (D) | 0.25 | 0.06 | 0.12 | 0.77 |
| I feel more and more engaged in my studies (D) | 0.45 | 0.34 | 0.11 | 0.73 |
| There are days when I feel tired before I arrive in class or start studying (E) (R) | 0.46 | 0.35 | 0.11 | 0.73 |
| After a class or after studying, I tend to need more time than in the past in order to relax and feel better (E) (R) | 0.46 | 0.34 | 0.11 | 0.73 |
| I can tolerate the pressure of my studies very well (E) | 0.51 | 0.41 | 0.10 | 0.72 |
| While studying, I often feel emotionally drained (E) (R) | 0.49 | 0.36 | 0.10 | 0.73 |
| After a class or after studying, I have enough energy for my leisure activities (E) | 0.42 | 0.28 | 0.11 | 0.74 |
| After a class or after studying, I usually feel worn out and weary (E) (R) | 0.51 | 0.40 | 0.10 | 0.73 |
| I can usually manage my study-related workload well (E) | 0.51 | 0.41 | 0.11 | 0.73 |
| When I study, I usually feel energized (E) | 0.45 | 0.33 | 0.11 | 0.73 |
| Test scale |  |  | 0.11 | 0.74 |
